# Mechanisims of asthma and allergic disease – 1072: CD48: a novel biomarker for asthma?

**DOI:** 10.1186/1939-4551-6-S1-P69

**Published:** 2013-04-23

**Authors:** Yael Minai-Fleminger, Amichai Gutgold, Ahlam Barhoum, Ron Eliashar, Vera Leibovici, Neville Berkman, Francesca Levi-Schaffer

**Affiliations:** 1 Pharmacology and Experimental Therapeutics, The Hebrew University, Jerusalem, Israel; 2 School of Medicine, The Hebrew University, Hadassah Medical Center, Jerusalem, Israel; 3 Dermatology, Hadassah University Medical Center, Jerusalem, Israel; 4 Institute of Pulmonology, Hadassah-Hebrew University Medical Center, Jerusalem, Israel

## Background

CD48, a CD2-family surface receptor expressed on immune cells involved in various immune disorders and cell activities has also a soluble form (sCD48) previously found to be elevated in the serum of leukemia and infectious diseases patients. We have demonstrated that human peripheral blood (pb) eosinophils express membrane-bound functional CD48 (mCD48) and that its engagement activates them in vitro and in vivo in a mouse model of asthma. In this study our hypothesis was that the levels of both mCD48 and sCD48 are increased in the pb of asthmatic and other allergic disease patients. Our aim was therefore to evaluate mCD48 expression on leukocytes as well as its soluble form in the serum of asthmatic, allergic rhinitis (AR) and atopic dermatitis (AD) patients.

## Methods

Pb from asthmatic (taking only inhaled bronchodilators), untreated AR and AD patients, and non allergic non asthmatic controls was collected.Leukocyte fraction was isolated and stained using Abs against CD48 andthespecific markers for each cell type (T cells, B cells, neutrophils, monocytes, eosinophils, basophils and NK cells). **Results** were analyzed by FACS. sCD48 was detected using a specific ELISA.

## Results

mCD48 expression in asthmatics was significantly increased on eosinophils as compared with healthy donors (p<0.001). Importantly this enhanced expression was also found to be significant on neutrophils (p<0.015) and monocytes (p<0.05) although at a lower extent. Interestingly on both AR and AD derived cells, CD48 expression was decreased in comparison to the control group. In the asthmatic group sCD48 levels were also significantly elevated (p<0.006 and 2 folds higher) as compared with the control, AR and AD groups. We also found that sCD48 can down regulate eosinophil activation carried out by heat killed *S.aureus* by significantly reducing the release of IL-8 (p<0.005).

## Conclusions

Our results suggest that both the mCD48 and sCD48 may serve as novel biomarkers for asthma. sCD48 may regulatethe allergic response and provide a new potential target for the suppression of asthma. Further research must be done in order to evaluate CD48 expression and release throughout the course of the different forms of asthma and its expression and function in the airways.

